# Experience of maintaining tooth brushing for children born with a cleft lip and/or palate

**DOI:** 10.1186/s12903-017-0412-3

**Published:** 2017-08-24

**Authors:** Yin-Ling Lin, Karen Davies, Peter Callery

**Affiliations:** 1 Division of Dentistry, The University of Manchester, JR Moore Building, Oxford Road, Manchester, M13 9PL UK; 2 Division of Human Communication, Development and Hearing, The University of Manchester, Ellen Wilkinson Building, Oxford Road, Manchester, M13 9PL UK; 3 Division of Nursing, Midwifery and Social Work, The University of Manchester, Jean McFarlane Building, Oxford Road, Manchester, M13 9PL UK

**Keywords:** Cleft lip and/or palate, Tooth brushing, Childhood oral hygiene, Dental care for children

## Abstract

**Background:**

Children with a Cleft Lip and/or Palate (CL/P) have been reported to have poorer oral health than those without the condition. The consequences for these children can be particularly problematic due to implications for future treatments. Tooth brushing is an important behaviour contributing to children’s oral health, but is under researched in the CL/P population. The aim of the study is to explore the experience of maintaining tooth brushing among children in the United Kingdom (UK) with a CL/P and their parents.

**Methods:**

Semi-structured interviews were carried out with twenty-two parents and sixteen children with a CL/P (5-11 years), recruited at a cleft centre in the UK. Thematic analysis was used for data analysis.

**Results:**

Three key themes were drawn from the qualitative data: first, parents of children with a CL/P generally had strong motivation to look after their children’s teeth but children’s motivation was inconsistent. Second, parents were primary enablers of children’s tooth brushing behaviour, often employing approaches adapted to their child’s characteristics to encourage tooth brushing. Third, a range of obstacles were encountered by parents and children in maintaining regular tooth brushing behaviours. They reported obstacles such as issues related to CL/P, ‘forgetting’ and childhood illness.

**Conclusions:**

The paper suggests that parents of children with a CL/P need support to enact their intention to maintain regular tooth brushing and prioritise tooth brushing within the context of demanding and dynamic family life.

## Background

Dental caries is one of the most common childhood diseases and is mostly preventable through developing good oral health behaviours such as brushing teeth with fluoride toothpaste and controlling sugar intake [1, 2]. Therefore, many studies aim to improve our understanding of the facilitators of, and barriers to, good oral health in childhood [3–8], with the intentions of informing the design of effective interventions to enable good oral health behaviours. The effectiveness of behavioural interventions in caries prevention, however, has been inconclusive. For example, a Cochrane review of randomised controlled trials based in primary school settings found insufficient evidence for the efficacy of behavioural interventions for reducing caries [9]. A limitation of these studies was that none of the interventions explicitly referred to behaviour change theory [9]. The majority of studies used the premise that providing information will lead to change in behaviour but did not refer to developing health behaviour alongside information provision. Therefore, the results of oral health behaviour interventions lack consistency [10]. Another review paper examined studies that implemented behavioural interventions to reduce childhood caries reported that outcomes were variable and concluded further research was recommended to understand the mechanisms underlying behaviour change in oral health [11].

Cleft lip and/or palate (CL/P) is a congenital anomaly affecting facial structure [12]. It appears as an opening in the lip and/or palate and is generally treated with surgery during the first year of life. Approximately 1 in 700 babies are affected by cleft lip and palate, with variation in relation to geographic origin and ethnicity [13–15]. It can affect a range of functions including speech, hearing and psychosocial health [12, 13] and therefore it may impose a burden on both child and family. Poor oral health amongst children born with a CL/P may compromise the effectiveness of, or even preclude, future orthodontic treatments or alveolar bone grafting. In 2012, the James Lind Alliance (a non-profit making initiative recommending research priorities in the UK), with the help of those affected by CL/P, identified caries prevention, as one of the ‘top 12’ priorities for cleft research [16]. This shows that caries prevention is an important topic for families of children with a CL/P. There is evidence that parents of children with a CL/P are motivated to seek treatments, which tends to be expressed as an obligation, in agreeing to a course of medical interventions to address the difficulties arising from CL/P [17]. We know much less about parents’ motivation to supervise tooth brushing as part of everyday oral health care. In the UK, specialist multi-disciplinary cleft teams provide specific information and advice about oral health care [12]. However, despite the motivation and access to additional information, children born with a CL/P have been reported to have poorer oral health than children without this condition [18–24]. It is therefore important to understand the problems children and parents experience in establishing and maintaining an adequate tooth brushing routine in order to design effective interventions to help enacting tooth brushing in everyday life.

## Methods

The current study was exploratory using a qualitative research design to investigate parents’ and children’s experience of caring for the teeth of children with a CL/P repaired in infancy. The study design drew upon ethnomethodology [25], as a theory and method to understand the way in which people make sense of their everyday life in a given social situation, through their discourse. Ethnomethodology is particularly useful to understand routine situations of everyday life and the ways in which people continuously interpret and reinterpret everyday life events [26]. Hence, it provided a framework to explicate the ways in which tooth brushing are carried out by children and their parents in everyday family context. Semi-structured interviews were carried out with children and their parents.

The study design, interview topic guides and data analysis were completed collaboratively by three qualitative researchers with previous experience of interviewing parents and children, and working with children with a CL/P (YL, KD and PC). In order to reduce researcher bias, each stage of the study was informed and monitored by an advisory group of researchers, dental practitioners, cleft specialists and parents of children with a CL/P. The study was approved by the West Midlands NHS Research Ethics Committee (Ref. 14/WM/1153).

### Recruitment

**Fig. 1:**
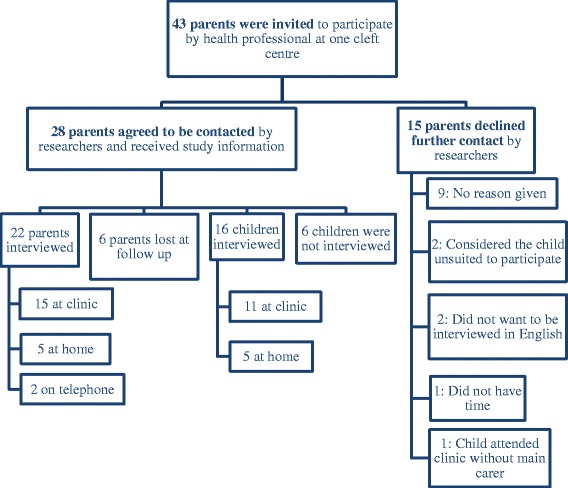
Summary of recruitment (May 2015–August 2015)

The inclusion criteria for this study were: children aged 5–11 years born with a CL/P and their parents. The age limits were selected to include children across the early stages of becoming independent in tooth brushing and those experiencing CL/P interventions such as orthodontic treatments and alveolar bone grafting. All children with a CL/P are scheduled for review in the UK between their fifth and sixth birthdays and some will continue to attend clinic appointments for further monitoring and treatments, which provided opportunity to recruit across this age group. Families undergoing significant psychosocial difficulties were excluded from this study. A purposive approach to sampling was also used to include variation in children’s age, gender and type of cleft. The recruitment process concluded when theoretical data saturation was reached, that is, the point at which no new themes or ideas emerged from additional participants [27]. Children with a CL/P and their parents were approached by a specialist dental health professional during their 5-year review or routine clinic appointments at a specialist cleft centre in the UK. Once the family agreed to take part in the study, two qualitative researchers (KD and YL) took written informed consent from the parents and verbal assent from the children. Fifty-one percent of those invited were interviewed (see Fig. 1).

### Data collection

Two qualitative researchers (KD and YL) carried out semi-structured interviews with parents and children at the cleft clinic (15 families), participants’ homes (5 families) or by telephone (2 families). Parents and children were interviewed on a one-to-one basis. They were given the options to be interviewed alone or with each other’s presence in the same room. All chose to be interviewed together, apart from those who opted for a telephone interview, where only parents were interviewed. Children were given the options to be interviewed before or after their parents. Most of them chose to be interviewed after their parents so that they could see what it was like to be interviewed. Interviews consisted of open ended questions about participants’ experience of looking after the teeth of children with a CL/P. The topic guides included questions concerning knowledge of oral health, beliefs and perceived obstacles and enablers for looking after teeth (Appendix 1). Children’s interviews included drawing activities and guessing games to relax children and promote initial conversations. A ‘story-telling’ framework was used to encourage children to construct a ‘story-line’ to integrate information into a coherent account (Appendix 2) [28]. The interviews were conversational in format with parents and children interjecting, corroborating or challenging each other’s account. This added to the richness of the data. The topic guide served the purpose of reminding researchers to cover the key issues but the wording and the order of the questions were guided by the flow of the conversation. Each interview took 10–29 min to complete. The topic guides and interview methods were tested with 2 parents and 4 children from two families, each consisting of one parent and two children, to ensure topic guides were appropriate and comprehensive prior to the fieldwork. All interviews were audio recorded and transcribed verbatim.

### Data analysis

Thematic analsysis was used to explore participants’ accounts to identify common themes in the data. It offers flexibility of being descriptive as well as interpretive [29]. The data were coded initially and then categorised into themes and subthemes [29]. The process was systematic but flexible to allow patterns to emerge from the data, without precluding exceptions. Data were divided into meaningful units but their connection to the context was maintained [30]. Following the ethnomethodological perspective, attention was given to the ways in which children with a CL/P and their parents accomplished, managed and reproduced the selected accounts of tooth brushing routines in everyday life [26]. Interview transcripts were systematically coded by two researchers (a lecturer in speech language and therapy KD and a lecturer in qualitative research methods YL) independently to increase the reliability of data analysis [31]. The codes were then compared and themes agreed by consensus amongst all authors (YL, KD and PC). A CL/P service user and a specialist dental health professional were invited to consider the themes identified and provide feedback, which was used to cross-examine the themes, providing confirmation or alternative interpretations of the themes. Participants’ identities have been removed in the following discussions and pseudonyms are used to ensure anonymity.

## Results

**Fig. 2:**
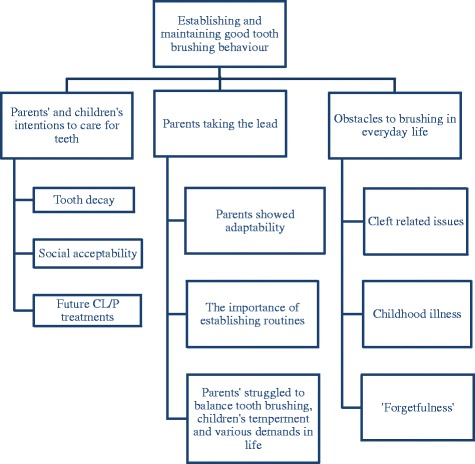
A summary of the results

Participants described everyday challenges and things that helped to care for the teeth of children with a CL/P. However, their accounts were not consistent with the terminology of ‘barriers’ and ‘facilitators’ used in previous studies of oral health. They did not describe permanent barriers to, or facilitators of, tooth brushing but indicated that everyday practices can vary in both improving practice and neglecting tooth brushing. These were more akin to ‘obstacles’ and ‘enablers’ that are open to change as circumstances change. A summary of the results is provided in Fig. 2 as follows:

### Parents’ and children’s intentions of caring for teeth

Some children with a CL/P, and particularly their parents, showed strong intentions to care for teeth. The intentions were mainly prompted by their concern about tooth decay, social acceptability and future CL/P treatments. Many parents talked about the importance of tooth brushing on preventing tooth decay. For example:

*I’ve always emphasised on it, because it’s always been, right, well, if you don’t want to brush your teeth then, A, they’re going to fall out, B, they’re going to go black and they’re going to fall out, or you’re not going to be able to chew your food. I always emphasise the consequences of not brushing your teeth. (Claire, mother).*

Claire, the mother of a 6-year-old girl with a CL/P, used terms such teeth falling out or having black teeth to describe tooth decay. These expressions were commonly adopted by other parents and children. For instance, eight-year-old Ben understood that tooth brushing ‘*cleans [teeth] and if you don’t clean then your teeth will rot*’. Children talked about the association between not brushing teeth and caries and they were, therefore, motivated to brush their teeth because they did not want their teeth to ‘*rot away*’ (Oscar) or get ‘*wobbly’* (Robin).

Many children and some parents referred to social acceptability, expressed concerns about cleanliness and how they would be perceived by others. Some children wanted to have a ‘*minty taste*’ so that ‘*you can draw a mouth with like nice air coming ou*t’ (Emilia, 11 years old). For them, social acceptability also included the colour of the teeth and the ability to speak and smile. The 9-year-old Jessica thought brushing teeth was important:

*Because when you smile, if you smile horrible rotten teeth no one will like your smile, but if you smile with nice clean white teeth people will like it. (Jessica, 9 years old)*

Some parents also cited social acceptability as their motivation to encourage their children to brush teeth. For instance, Rachel, the mother of a 10-year-old boy with a CL/P, told their children to ‘*go and brush your teeth! Because you don’t want to have smelly breath!*’ In addition, parents expressed concerns about future CL/P treatments as contributing to a strong intention to look after their children’s teeth. The following mother, Alice, said she brushed the teeth for her child, explaining:

*We’ve just talked more about how he’s going to need braces and things, so that’s another reason for keeping on top of it. I think it’s been more motivating for us than for the others (who do not have CL/P). (Alice, mother)*

Lucy, the mother of a 7-year-old girl with a CL/P, was motivated to look after all of her children’s teeth, but distinguished her child with a CL/P because of her ‘problems’:

*Well, I want them to have nice teeth, that’s the most important thing for me, you know, and have a healthy mouth more than anything, her more than my son, of course, because of her problems but yes… (Lucy, mother)*

All parents interviewed expressed strong and consistent intentions to look after their children’s teeth. Some children also reported firm intentions to carry out tooth brushing routine, but others were less engaged. Children’s intentions were more closely associated with social acceptability and none of them referred to the impact of oral health can have on future CL/P treatments. By contrast, parents tended to identify tooth decay and future CL/P treatments as their main motivation to care for their children’s teeth.

### Parents taking the lead

Children regarded their parents as the main enabler of caring for their teeth and parents recognised that they needed to take the lead, although they varied in how closely they supervised their children. Parents’ roles were described as teachers, reminders, supervisors and enforcers by themselves and their children. They demonstrated adaptability in encouraging children to brush teeth. For instance, Helen, the mother of a 5-year-old boy with a CL/P, reported encouraging tooth brushing by ‘*singing and tickling and praising*’. When this was not successful, she became more direct, stating that she would ‘*just pin him [down] and do it anyway*’. In this instance, the parent was explicit that the choice of the strategy was determined by the child’s ‘*mood*’. Furthermore, parents highlighted the importance of establishing routines in encouraging children’s tooth brushing. For example, the following mother, Anne, described the way in which routine helped her child to remember tooth brushing.

*Yeah, routine is just the key of getting up, having his breakfast, getting dressed, washing, brushing his teeth, going to school. So he knows it’s all in his routine. Sometimes he’ll say to me, it’s time to brush my teeth, you know, because it’s that scheduled. (Anne, mother)*

Some parents talked about the tooth brushing routine involving themselves or other family members. This was said to also encourage the tooth brushing behaviour of the child with a CL/P. In the following example, Claire described the morning tooth brushing routine in their household:

*We brush at the same time. It’s part of our routine. We’ll get dressed, we’ll do her hair, I’ll get my clothes on, we’ll go to the bathroom together, we’ll brush together and then we’re off then. (*Claire*, mother)*

The following father talked about the child with a CL/P brushing with his sister so they encourage each other’s brushing behaviour or ‘egg each other on’.

*It’s kind of like egging each other along type of thing. She does exactly the same thing [as him]. She didn’t today, but she’s done it before where she’ll go off and do her teeth and she’ll go [Max, the child with a CL/P] you’ve not done your teeth. Then she’ll bring his toothbrush and his egg timer […] if he walks off she’s followed him. [She would say] I'm making sure that you're doing it properly. Then she'll come up to me and go, with a full brush of toothpaste, he's not brushing his teeth.* (*Martin, father)*

Ten-year-old Oscar’s parents in the following example described a monitoring role, asking Oscar if he had brushed his teeth and listening for the sound of the electric toothbrush.

*With him, he's not a very good liar. He would try and try and try so we'd stand there and go, think about it. Did you brush your teeth? Because I'll open your mouth and check it. Then he'll go, let me just go and check. Then he'll go and do it. (Hannah, mother)*

*At the moment he's using one of those vibrating toothbrushes as well, so if you don't hear it, we usually check the sink as well. So if there is a large deposit of - so usually if he's done it quite well it's just very watery or frothy and stuff like that. If he hasn't then he's essentially just spat out toothpaste and stuff. (Martin, father)*

Some parents in this study expressed challenges in balancing doing the right thing and preserving harmony in the family as some children were described as being disinterested or reluctant to brush teeth. The challenges parents faced are illustrated by Anne, who sometimes struggled to motivate her child, Robin, to brush his teeth:

*We do have traumas like them running round the table and they’re jumping over settees and, urgh, it’s not time to brush my teeth yet. (Anne, mother)*

Anne thought it was a natural reaction that her child was reluctant to brush because ‘he’s a child’. As the main enabler, parents found it challenging to balance tooth brushing, their children’s temperament and the various demands of family life.

### Obstacles to children’s tooth brushing behaviour

Children and parents mentioned treatments and misaligned teeth related to CL/P as posing challenges for maintaining a good oral health routine. For instance, 9-year-old Georgina explained that bone graft surgery made tooth brushing more difficult.

*Interviewer: Right, so can you remember what was the reason that stopped you doing your teeth [after the bone graft surgery]? Only tell me, because I’ve never had one.*

*Georgina: ‘Cause it hurt.*

*Interviewer: It hurt did it? Yeah. And did it actually hurt even to put a toothbrush in your mouth?*

*Georgina: It even hurt when I opened it.*

The following mother of a 9-year-old girl with a CL/P described taking responsibility for brushing her child’s teeth for the previous six months because her child found it difficult to brush awkwardly growing teeth.

*I started brushing them myself, it’s been about six months I think, because she wasn’t quite doing it properly herself and she found it difficult to get in all…you know, like especially when all these teeth start showing at different angles, she found it difficult to brush herself.(*Rani*, mother)*

Some children, like Noel, have a sensitive mouth that might be related to CL/P and brushing can cause pain and this can also lead to resistance in tooth brushing.

*[He just says his mouth’s sore, his teeth hurt […] he struggles expressing and telling me how he feels or he gets everything muddled up a bit and stuff, so he does struggle telling you, he just says he doesn’t like it and it hurts (Helen, mother).*

Some parents reported that tooth brushing behaviour can be interrupted when children felt ill. Both long-term and short-term illnesses were presented as factors beyond parents’ control and described as inevitable obstacles to caring for teeth.

*Well, she had a bug, so she was being sick all the time, and if she wasn’t sleeping she was burning up. She couldn’t move off the couch. This went on for about a week. So the last thing she wanted to do was go to the bathroom and brush her teeth. (Kirsty, mother)*

‘Forgetting’ was also referred to by children and parents as an obstacle, either to brushing teeth or reminding their child to brush. When participants were asked why they forgot or why they found it difficult to remember, they referred to time constraints in the mornings and children’s tiredness in the evenings. The term ‘forgetting’ was sometimes used to describe conscious decisions to miss tooth brushing as well as failures of memory. This could be in response to managing routine challenges such as being late for school, or a child’s fatigue. For example, 9-year-old Jessica’s account below illustrates ‘forgetting’ to brush her teeth when she was in a rush in the morning:

*[…] when I’m late for school I’d miss the register or I don’t want to miss assembly, because sometimes we have this thing called green cushion, and it’s when you get, like, a cushion for being good that you can sit on, and then, like, if you get it you don’t want to be late to anything. (Jessica, 9 years old)*

Given the option of brushing teeth or being late for school, resulting in missing the register and subsequently losing the ‘green cushion’, brushing was described as something that could be traded-off and missed for the day. Some parents responded to questions about forgetting in a confessional tone, with a morally laden response as seen in Rani ‘s comment about allowing her child to miss brushing: ‘I’m not going to lie’:

*Once or twice I have [allowed my child to miss brushing]. I’m not going to lie and say no, I haven’t. I have once or twice, I’ve said okay, but I still tell her but just give it a good rinse if you don’t want to do that two minutes. (Rani, mother)*

This may reflect the concerns parents had for doing their best to look after their children and presented themselves as only occasionally allowing their children to miss tooth brushing.

## Discussion

We have presented three observations derived from the data: first, some children with a CL/P and all parents showed a strong intention in making sure tooth brushing takes place. Second, as children’s intentions were inconsistent, parents assumed responsibility to care for their children’s teeth. Parents had to be adaptable to maintain daily tooth brushing. This was described as adapting to their children’s behaviour on a day by day basis. This was often helped by having a regular routine that children could follow. Nevertheless, parents found it challenging to balance doing the right thing and keeping a harmonious family in the context of demanding family life. Finally we reported the obstacles to tooth brushing in everyday life, including difficulties directly related to CL/P and other childhood illness. Moreover, ‘forgetting’ was identified as one of the main obstacles in maintaining children’s tooth brushing behaviour. However, when children and parents talked about ‘forgetting’ to brush teeth, some of them were referring to a conscious decision not to brush teeth for reasons they saw appropriate at the time in their family life.

Policy and academic research have highlighted the merit of educating parents about the importance of dental health and the best ways to care for their children’s teeth [5, 6, 32–36]. Nonetheless, this study has shown that whilst educating parents about dental health is important in improving children’s oral health, it does not appear to be sufficient. Parents of children with a CL/P understand the importance of oral health, expressed intentions to care for teeth and have received additional information about oral health care throughout their child’s early life. Furthermore, they are knowledgeable in looking after their children’s teeth [37]. The current study showed that establishing and maintaining tooth brushing routines is complicated by a range of factors common to families, together with specific issues associated with CL/P. Some children can have discomfort or experience difficulties in brushing for reasons associated with CL/P or treatments. Many parents demonstrated their adaptability and intentions to encourage tooth brushing but also reported obstacles in everyday life interfered with tooth brushing routine. These obstacles can undermine their intentions to maintain their children’s oral health, suggesting that an intention-behaviour gap exists [38]. Although ‘forgetting’ to brush teeth has been reported as one of the obstacles, some families used forgetting to describe a conscious decision to miss tooth brushing to manage the challenges in everyday life, indicating that intentions might be difficult to enact due to various demands in family life. Therefore, it is important to challenge the assumption that tooth brushing will automatically take place with sufficient intentions and knowledge. The experience of maintaining tooth brushing routine reported by children and parents demonstrated the complexity of challenges families encountered in their endeavour, which can also affect the relationship between parents and children. Consequently, families often take a pragmatic approach to maintain tooth brushing. For example, establishing tooth brushing as part of the daily routine provided an agreed expectation for children and parents. However, the routine can sometimes be disrupted when required. The change of the routine will be negotiated by parents and children.

The evidence from children with a CL/P and their parents suggests that in addition to providing information about how to maintain oral health, extra support may be required to help parents and children to implement their intention and knowledge in their own family context. This might include guidance to identify obstacles, opportunity to consider ways of overcoming them and techniques to enact intentions. Interventions aiming to change oral health behaviour must harness intentions and support parents and children to translate their intentions into changes in health behaviour. A further study is currently taking place to explore the benefits of an implementation intentions intervention [39] with children with a CL/P and their parents.

The current study focused on children with a CL/P but many of the obstacles reported were not specific to children with a CL/P. Moreover, some enablers and obstacles in everyday family life reported in the current study maybe relevant to other everyday childhood health behaviours. We hope that some elements of our findings can also be of use to inform studies investigating oral health and in other populations.

## Strengths and limitations

Three points should be considered in the interpretation of the findings of this study: first, more than half of the approached participants agreed to be involved in this study. This suggests that they were motivated to share their experience with researchers. Hence, those who did not feel comfortable or unwilling to talk about their tooth brushing practices might have been inevitably excluded from the study. We have, however, included a group from reasonably diverse demographic backgrounds, across different cleft types, child’s age and gender. Hence, we have reasonable confidence in the range of views captured in the study. Second, participants were recruited from one cleft centre in the UK. Therefore, caution should be used in applying the findings to other contexts. Finally, all children in this study chose to be interviewed with their parents, which may have influenced the responses given [40]. Although it is inevitable that the presence of the interviewee’s parents or children might influence what was said in the interview, it was also observed that parents and children often challenged each other’s accounts. This can be seen as an attempt to construct an account that reflects their daily practices. These collectively produced accounts may potentially enhance the quality of the data.

## Conclusions

This paper reports the challenges that children with a CL/P and their parents in maintaining regular tooth brushing but also describes the intentions that they have to ensure tooth brushing takes place. We suggest that parents and children with a CL/P could benefit from support to enable them to translate intentions into maintaining regular tooth brushing and prioritise tooth brushing in the context of demanding family life.

## Appendix 1

### Topic guide for interviews with parents

**Section 1: Dental care routines**

**Tooth brushing**

1. What do you do to look after your children’s dental health? What kind of things do you do differently for your child with CL/P if at all?
2. Can you describe your child’s tooth brushing routine
  - Who usually brushes their teeth?
  - when, how often, how long, type of brushes, type of toothpaste,
  - Is it automatic or prompted: how do you remind your child?
  - When (at what age) do you think your child understood that cleaning their teeth was important?
  - How does it link with other daily routines?
  - Parents’ role-how do you check how well they are doing?
3. How did your child learn about tooth brushing? What has been most useful in encouraging your child’s tooth brushing? Why?

**Drinks and snacks**

4. Can you describe your child’s usual drinks and snacks? When does he/she have them?
5. How much difference do you think this makes to the care of his teeth-what would your child say?
6. Who chooses the drinks and snacks?

**Dental treatment**

7. What difference does it make when your child goes to the dentist?
8. What does your child think about going to the dentist?
9. How often do they go/ Who do they see?

**Section 2: Barriers and facilitators**

10. How would you sum up your role in supporting your child’s dental care? Who takes charge of looking after your child’s teeth
11. Do you or your child sometimes forget about looking after his teeth?
12. What kind of support have you and your child had to help them with dental care? Is there anything you would have changed?
13. What is the most important in helping your child look after his/her teeth?

**Section 3: Views about future study**

14. Would you be happy to participate in any one of the groups? Reasons?
15. What information would parents want before they agree to allow their child to take part
16. How would you like the information about the study presented-verbal, written or other means?
17. How would be the best way to ask parents to be involved-email/post/face to face?

**Any other comments and thanks**

## Appendix 2

### Topic guide interviews with children

**Section 1: Looking after your teeth**

*Let’s start with a picture-this is me; you can draw yourself. Here’s a story framework so we can draw what we do when we look after our teeth.*

What do you usually do to look after your teeth? [who, how often, when?].

What helps you look after your teeth? What helps you the most?

How do you remember to brush your teeth? [routines/other activities].

So tell me exactly what you do when you clean your teeth?

What kind of things get in the way of you looking after your teeth?

How do you feel about brushing your teeth?

**IPad speech bubbles activity.** *Let’s look at this boy-he might need your help.*

How would you help this boy look after his teeth?

What kind of problems do you think he has with his teeth? [Why?]

Why do you think that would help him?

Can you think of anything that would help him?

*Let’s tell him what to do using speech bubbles*

- ▪ What should he do?
- ▪ What problems might he have
- ▪ What makes it easy or difficult?
- ▪ How would you help him?
- ▪ What do think helps you the most?

*Can you make up a way to help him remind himself.*

Visiting dentist – purpose, own experiences.

**Section 2: Snacks and drinks**

What are you favourite treats and snacks and drinks?

How often do you have them?

How do you get your snacks and drinks?

**Picture activity** *This boy is going away to stay with his gran and he’s deciding what snacks and drinks to take.*

What treats do you think he should take? Why?

Will it make any difference to his teeth? In what way?

Are there good times to eat these treats?

Could you make up a motto to help him remember how to look after his teeth when he’s away?

## Abbreviations

CL/P: Cleft Lip and/or Palate
